# Identifying genetic determinants of *Mycobacterium tuberculosis* acid growth arrest by transposon mutagenesis coupled with next-generation sequencing (Tn-seq)

**DOI:** 10.1128/mra.00060-25

**Published:** 2025-07-11

**Authors:** Claire Healy, Thomas R. Ioerger, Sabine Ehrt, Alexandre Gouzy

**Affiliations:** 1 School of Medicine, Trinity College Dublin 8809, Dublin, Ireland; 2 Department of Computer Science and Engineering, Texas A&M University 14736, College Station, Texas, USA; 3 Department of Microbiology and Immunology, Weill Cornell Medical College 12295 , New York, New York, USA; University of Maryland, Baltimore, Maryland, USA

**Keywords:** acid stress, transposon library, *Mycobacterium tuberculosis*

## Abstract

*Mycobacterium tuberculosis,* the causative agent of tuberculosis, remains the leading global infectious disease killer. Adaptation of *Mycobacterium tuberculosis* to acidic niches within the host during infection is vital to establish the disease. Here, we present a high-density transposon mutant sequencing library data set identifying genetic determinants of acid growth arrest to serve as a resource.

## ANNOUNCEMENT

Tuberculosis (TB) remains a leading global infectious disease despite the availability of a vaccine and antibiotic treatment. *Mycobacterium tuberculosis* (Mtb), the causative agent of TB, has evolved to adapt and withstand many stresses, including acid stress, during infection (1). However, the mechanisms controlling Mtb replication and survival during host-imposed stress remain ill-defined. We recently published a study implicating phosphate sensing and uptake in regulating Mtb growth in acid, for which this article is a companion (2). We performed a transposon sequencing (Tn-seq) screen using an Mtb transposon mutant library to identify genes which, when interrupted, alter Mtb fitness during acid stress, providing a transposon insertion abundance data set presented here.

A saturated transposon mutant library was previously constructed in *M. tuberculosis* H37Rv, achieving at least 60% coverage of the TA dinucleotide insertion sites in the genome as described previously (3, 4). Briefly, a 100 mL culture of wild type *M. tuberculosis* H37Rv strain was grown to optical density of ~1 at 37°C. The bacteria were pelleted by centrifugation, washed, and incubated with the ɸMycoMarT7 phage at a multiplicity of infection of 2 for 4 hours at 37°C. After transduction, the bacteria were washed, cultured on solid media containing Tween-80 and kanamycin, and incubated for 3 weeks at 37°C. The library of ~10^5^ mutant colonies was harvested by scraping and stored as frozen −80°C stocks. The library coverage of TA dinucleotide sites was confirmed to be at least ~60% by Illumina sequencing.

To identify genetic determinants of acid growth arrest in Mtb, the transposon mutant library was cultured on Middlebrook 7H10 agar supplemented with 0.5% (v/v) glycerol and Middlebrook OADC supplement with the final pH adjusted to 7.0 or 5.5. Ten 15 cm plates were used for each condition, and the library density was aimed at 10^5^ colonies/plate. The mutant library plates were scraped to harvest the bacteria after 3 weeks (pH 7.0) or 6 weeks (pH 5.5) incubation at 37°C. The experiment was performed three times independently.

To quantify the composition of the mutant transposon libraries after selection on acidic or neutral pH media, genomic DNA was extracted and isolated from the harvested libraries. Bacterial cells were disrupted enzymatically with lysozyme overnight, followed by incubation with SDS, proteinase K, and RNase. Cetyltrimethylammonium bromide in high salt conditions was used to precipitate cell wall components. The DNA was then further purified by phenol-chloroform-isoamyl extraction, precipitated using isopropanol, washed with ethanol, air dried, and resuspended in DNase-free water. The DNA libraries were prepared as follows: DNA was sheared to 500 bp using a Covaris M220 Focused Ultrasonicator, followed by end repair and addition of A-tails using NEB Next Ultra End Repair/dA-Tailing Module. DNA was purified using a Qiagen Polymerase chain reaction (PCR) purification kit, and overnight ligation of random barcoded adaptors was performed using NEB T4 DNA ligase. The DNA libraries were then purified using Agencourt AMPure XP magnetic beads (3). The transposon-chromosomal junctions were then amplified by PCR (Choice Taq polymerase), and the resulting DNA was then size selected to 300–400 bp using a Pippin Prep (Sage Science) as per the manufacturers’ instructions. Further junction amplification, as well as the addition of sequences required for Illumina sequencing, was performed by hemi-nested PCR (Choice Taq polymerase) (3). The DNA was finally purified, quantified, and checked for quality using an Agilent Bioanalyzer System. DNA libraries were then sequenced using Illumina’s Next-Generation Sequencing Technology (Macrogene). 100 bp paired-end sequencing was performed on an Illumina HiSeq 2500 platform, targeting a minimum sequencing depth of 300 million reads/run. Total reads of samples ranged from 9.5 million to 14.9 million, giving a coverage ranging from 125 to 200 reads per TA site. Quality of reads was assessed by Phred quality score (q-score). The percentage of reads in each sample library with a q-score >30 (99.9% base call accuracy) was at least 89.3%.

Raw sequence data (Accession number: PRJNA935700) were processed using the Transit Pre-Processor (TPP) tool in the TRANSIT Tnseq analysis software (5). Reads (in triplicate) were mapped to the H37Rv genome sequence (Accession no: NC_018143.1) using the Burroughs Wheeler Aligner (6) in TRANSIT.

**Fig 1:**
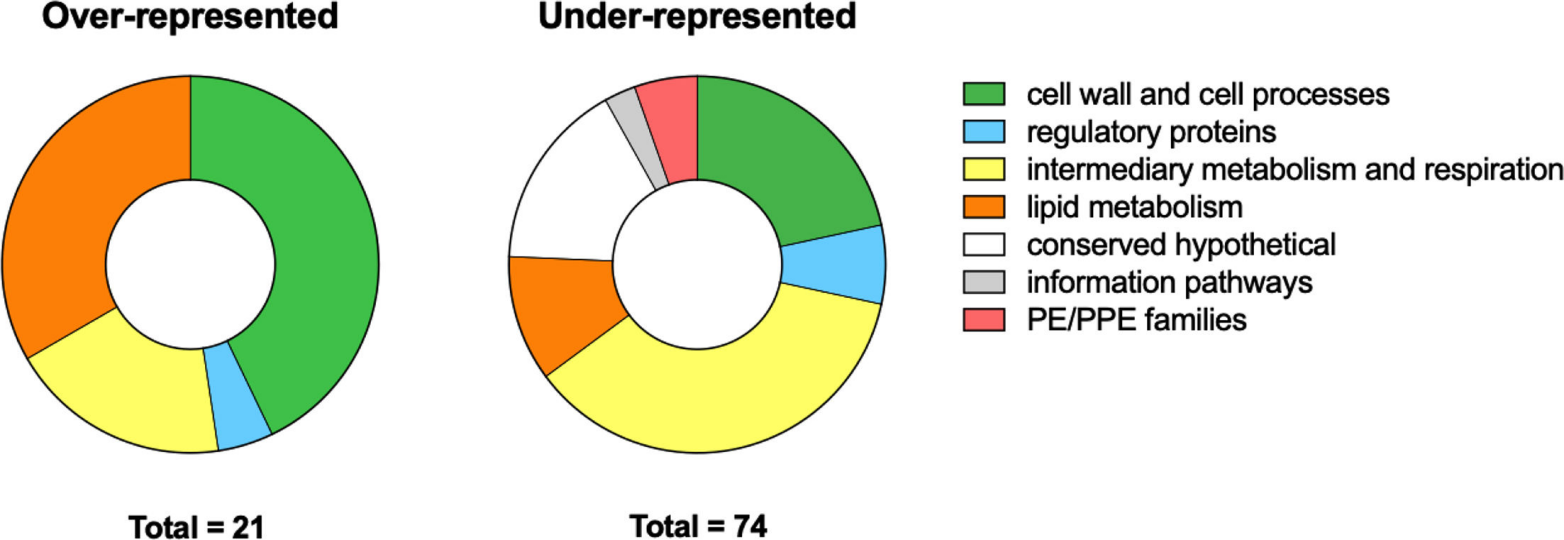
Functional categories of genes for which interruption significantly alters Mtb fitness during acid growth. Pie charts representing the total hits (= 95) comprising 21 over-represented and 74 under-represented hits sorted by functional categories (color coding, see legend).

Our data set identified insertions in 95 genes resulting in significantly altered fitness in acidic conditions compared to neutral pH; 21 mutants were over-represented in the library (fitness advantage) and 74 were under-represented (fitness defect) (Fig. 1). A selection of single gene knockout mutants validated the Tn-seq data set (2). Genome-wide mutant screening data sets are crucial tools to enhance our understanding of Mtb pathogenesis and physiology. Our TnSeq sequencing data set will serve as a resource to enable investigators to identify the impact of gene interruptions on the fitness of Mtb under acid stress, a host-relevant stress.

## Data Availability

The raw sequencing data are available on the NCBI Bioproject archive https://www.ncbi.nlm.nih.gov/bioproject/ under the following accession number: PRJNA935700. The file containing the list of insertions at each genetic loci and genes with significant over- or under-representation within the libraries after culture on pH 5.5 media (Data Set S1 - mbio.02825-24-85s0001.xlsx) is available as supplemental data to our original article (2) at https://journals.asm.org/doi/10.1128/mbio.02825-24.
